# Targeting CD46 Enhances Anti-Tumoral Activity of Adenovirus Type 5 for Bladder Cancer

**DOI:** 10.3390/ijms19092694

**Published:** 2018-09-10

**Authors:** Manh-Hung Do, Phuong Kim To, Young-Suk Cho, Se-Young Kwon, Eu Chang Hwang, Chan Choi, Sang-Hee Cho, Sang-Jin Lee, Silvio Hemmi, Chaeyong Jung

**Affiliations:** 1 Department of Anatomy, Chonnam National University Medical School, Gwangju 61469, Korea; manhhung.cnsh@gmail.com (M.-H.D.); tkphuong2609@gmail.com (P.K.T.); tonytoy@hanmail.net (Y.-S.C.); candy8900@naver.com (S.-Y.K.); 2 Institute of Genome Research, Vietnam Academy of Science and Technology, Hanoi 122121, Viet Nam; 3 Department of Urology, Chonnam National University Medical School, Gwangju 61469, Korea; urohwang@jnu.ac.kr; 4 Department of Pathology, Chonnam National University Medical School, Gwangju 61469, Korea; cchoi@jnu.ac.kr; 5 Department of Hemato-Oncology, Chonnam National University Medical School, Gwangju 61469, Korea; shcho@jnu.ac.kr; 6 Genitourinary Cancer Branch, Research Institute of National Cancer Center, Goyang 10408, Gyeonggi-do, Korea; leesj@ncc.re.kr; 7 Institute of Molecular Life Sciences, University of Zurich, Zurich 8057, Switzerland; silvio.hemmi@imls.uzh.ch

**Keywords:** CD46, CAR, adenovirus, gene therapy, bladder cancer

## Abstract

CD46 is generally overexpressed in many human cancers, representing a prime target for CD46-binding adenoviruses (Ads). This could help to overcome low anti-tumoral activity by coxsackie-adenoviral receptor (CAR)-targeting cancer gene therapy viruses. However, because of scarce side-by-side information about CAR and CD46 expression levels in cancer cells, mixed observations of cancer therapeutic efficacy have been observed. This study evaluated Ad-mediated therapeutic efficacy using either CAR-targeting Ad5 or CD46-targeting Ad5/35 fiber chimera in bladder cancer cell lines. Compared with normal urothelia, bladder cancer tissue generally overexpressed both CAR and CD46. While CAR expression was not correlated with disease progression, CD46 expression was inversely correlated with tumor grade, stage, and risk grade. In bladder cancer cell lines, expression levels of CD46 and CAR were highly correlated with Ad5/35- and Ad5-mediated gene transduction and cytotoxicity, respectively. In a human EJ bladder cancer xenograft mouse model, with either overexpressed or suppressed CD46 expression levels, Ad5/35-tk followed by ganciclovir (GCV) treatment significantly affected tumor growth, whereas Ad5-tk/GCV had only minimal effects. Overall, our findings suggest that bladder cancer cells overexpress both CAR and CD46, and that adenoviral cancer gene therapy targeting CD46 represents a more suitable therapy option than a CAR-targeting therapy, especially in patients with low risk bladder cancers.

## 1. Introduction

According to estimates by the American Cancer Society, approximately 81,190 people will be diagnosed in 2018 with bladder cancer, and approximately 17,240 people will be died from this disease in the United States [1]. Bladder cancer is the most frequent cancer found in the urinary system, and the fourth most common cancer in men. Although it is more common in males, bladder cancer is also prevalent in females. Currently, several strategies have been used to treat bladder cancer such as surgical resection, chemotherapy, immunotherapy, or irradiation. However, the poor survival rate and high frequency of recurrence require alternative and more efficient strategies [2,3,4]. Over the last few decades, viral cancer gene therapy has been suggested as an effective alternative therapy for controlling cancer, in particular for the treatment of bladder cancers [3,5].

Adenovirus (Ad) vectors are the most commonly employed vectors for cancer gene therapy. Ad gene therapy has shown good anti-tumor activity and immune tolerance, and, accordingly, the number of clinical trials using this virus has increased. Over recent years, more than 500 gene therapy trials have been or are being conducted with human Ad vectors [6], most of which include treatment of cancers [7]. As of now, there are about 57 serotypes of Ads that have been identified in human, classified into seven species (from A to G) [7]. Among the applicable serotypes, species C Ad5 is the most frequently used adenovirus in both experimental and clinical settings [7,8,9]. Ad5 has been demonstrated to mainly bind to the coxsackie-adenovirus receptor (CAR). However, the low expression of CAR in cancer cells has limited the efficiency of Ad5-mediated cancer therapies [10,11,12,13]. Alternatively, species B Ads, which utilize several host cell receptors including CD46 (Ad3/11/35) and desmoglein-2 (Ad3/7/11/14), have been employed to provide more effective cancer targeting [10,14,15,16,17,18]. In fact, we have previously shown that in colon cancer cells, CD46 effectively mediated gene transduction of an Ad5 fiber chimeric virus modified to contain the fiber knob from species B Ad35 [19].

CD46 belongs to the membrane cofactor protein (MCP) family that is involved in the regulation of complement activation, and is expressed in all nucleated cells. The main function of CD46 is to protect cells from complement-mediated cell lysis [20]; in addition, CD46 also works as a receptor for species B adenoviruses, as well as other viral and bacterial pathogens [21]. Expression of CD46 is low in normal cells, but is upregulated in human cancer cells such as ovarian cancer, breast cancer, lymphoma, hepatocellular carcinoma, lung cancer, prostate cancer, and colon cancer, presumably to protect the cancer cells from the complement system [19,22,23,24,25,26,27]. Overexpression of MCP members including CD46 was also found to lead to resistance to antibody-mediated complement activation targeting cancer cells [28]. Therefore, Ad-based cancer gene therapies targeting overexpressed CD46 have been suggested to enhance the antitumor capability, and to improve the gene transduction efficiency [29,30,31,32,33,34,35,36].

Although several viral cancer gene therapy strategies have been evaluated in bladder cancer models both in vitro and in vivo [5], gene therapy approaches based on adenovirus still require more detailed studies in order to improve their therapeutic efficacies [37]. In this study, we first correlated the expression of both CAR and CD46 to clinic-pathological features of bladder cancers. We then performed in vitro and in vivo experiments using several human bladder cancer cell lines, demonstrating that for these cells, a CD46-targeted Ad cancer gene therapy is much more effective than a CAR-targeted therapy.

## 2. Results

### 2.1. CD46 Expression Is Inversely Correlated with Bladder Cancer Progression

**Figure 1:**
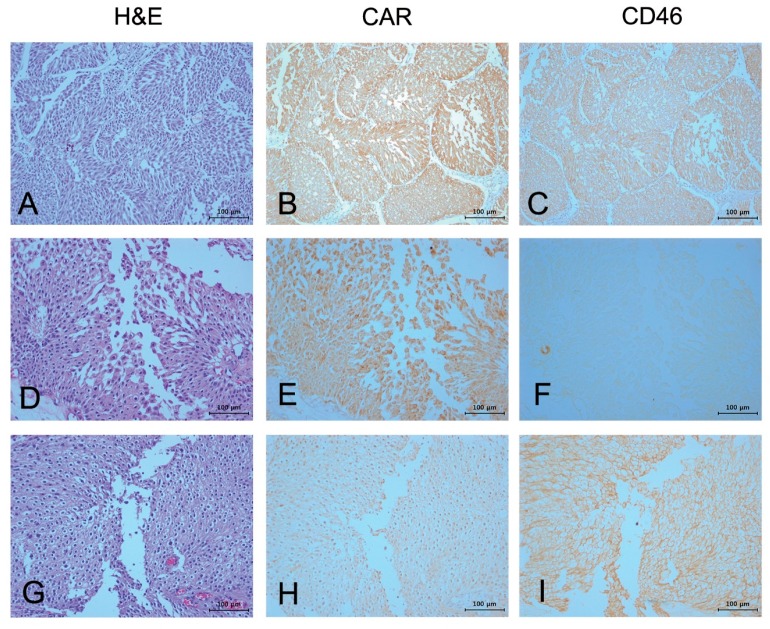
Immunohistochemical analysis of CD46 expression in bladder cancer patient samples. Serial sections of the selected bladder cancer tissues were immunostained with either antibodies to coxsackie-adenoviral receptor (CAR) (**B**,**E**,**H**) or CD46 (**C**,**F**,**I**). While some tumors express both CAR and CD46 (**A**–**C**), others express either CAR (**D**–**F**) or CD46 (**G**–**I**) only. The scale bar represents 100 μm. file attached.

**Figure 2:**
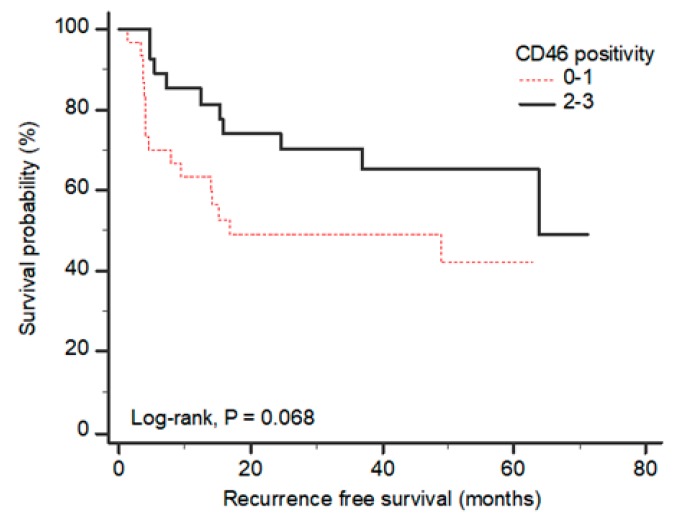
High CD46 expression indicates better survival of bladder cancer patients. Overall survivability was demonstrated by the Kaplan–Meier curve and measured by the log-rank test (*p* = 0.068).

Fifty-nine bladder cancer tissue samples from patients with clinical records were evaluated for their expression of CD46 and CAR by immunohistochemistry. Samples were scored by two investigators, including a pathologist. While the expression of both CAR and CD46 was very low in normal urothelium (data not shown), tumor cells derived from the bladder epithelium showed mixed results. While some tumors highly expressed both CAR and CD46 (Figure 1A–C), other tumors expressed only one protein or the other (Figure 1D–I). As a result, CAR expression was observed in 37 bladder tumors out of 59, whereas CD46 was observed in 29 bladder tumors. The expression data for each protein were statistically evaluated for their correlation to the clinico-pathological features of the bladder cancer patients (Table 1). While CAR expression was not correlated with disease progression (data not shown), CD46 expression was negatively correlated with tumor stage (*p* = 0.017), tumor grade (*p* = 0.012), and European organisation for research and treatment of cancer (EORTC) risk group (*p* = 0.042). However, multifocality, concomitant carcinoma in situ (CIS), intravesical chemotherapy, and recurrence did not statistically correlate with CD46 expression. In addition, the overall survival of bladder cancer patients tended to correlate with CD46 expression during a follow-up study up to 72 months (*p* = 0.068 by the log-rank test) (Figure 2). There was no correlation in the co-expression of CAR and CD46 in tumor samples and clinico-pathological features (data not shown). These results suggest that both CAR and CD46 are highly expressed in bladder cancers. CD46 was especially overexpressed in low grade and low stage cancers, whereas its expression was reduced in later stage cancers.

### 2.2. CAR and CD46 Promote the Gene Transduction Efficacy of Ad5 and Ad5/35 Respectively Confirmed

**Figure 3:**
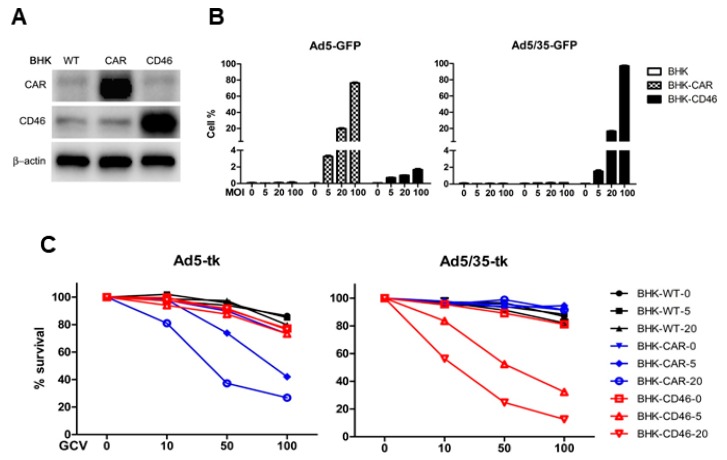
Gene transduction efficacy of adenovirus (Ad)5/35 is enhanced in CD46-expressing cells. (**A**) Western blot analysis of CAR and CD46 expression in parental rodent baby hamster kidney (BHK) cells or BHK-CAR and BHK-CD46 cells, which ectopically express the Ad receptors CAR and CD46, respectively. (**B**) Flow cytometry analysis of Ad-mediated green fluorescent protein (GFP) expression in BHK, BHK-CAR, and BHK-CD46 cells. Transduction of GFP by Ad5 (left panel) or Ad5/35 (right panel) analysis was measured by flow cytometry. (**C**) A cell killing assay was performed for all three BHK cell lines using either Ad5-tk (left panel) or Ad5/35-tk (right panel) followed by ganciclovir (GCV) treatment. Cytotoxicity was analyzed by the 3-(4,5-dimethylthiazol-2-yl)-2,5-diphenyltetrazolium bromide (MTT) assay. Error bars represent standard error (SEM). Statistics: C, *p* < 0.01 by two-way analysis of variance (ANOVA). Confirmed.

To investigate the efficacy of either CAR or CD46-mediated adenoviral gene transduction, baby hamster kidney (BHK) cells, with low endogenous expression levels of CAR and CD46, were used. The BHK cells were stably transfected to express CAR or CD46 [10] (Figure 3A). The overexpressing clones were tested for their transduction efficacy by CAR-binding Ad5-green fluorescent protein (GFP) and CD46-binding Ad5/35-GFP using flow cytometry analysis. Ad5/35 is a chimeric virus, in which the fiber knob domain of the Ad5 capsid has been replaced with the fiber knob from species B Ad35, as previously described [19]. A fluorescence-activated cell sorting (FACS) analysis at 24 h after viral infection revealed significant dose-dependent increases in the transduction efficiencies of Ad5-GFP and Ad5/35-GFP in BHK-CAR and BHK-CD46 cells, respectively (*p* < 0.01 by two-way analysis of variance (ANOVA)) (Figure 3B). We also tested the cytotoxic effect of adenoviruses expressing the pro-drug activating thymidine kinase (tk) in presence of ganciclovir (GCV), as previously described [19]. Cells were infected with Ad5-tk or Ad5/35-tk (5–20 multiplicities of infection (MOI)) and treated with GCV (10–100 μg/mL). After four days, the 3-(4,5-dimethylthiazol-2-yl)-2,5-diphenyltetrazolium bromide (MTT) proliferation assays demonstrated that Ad5/35-tk and GCV had a dose-dependent cytotoxic effect only on BHK-CD46 cells (*p* < 0.01 by two-way ANOVA) (Figure 3C). On the other hand, the cytotoxic effect of Ad5-tk/GCV was limited to BHK-CAR cells. These results confirmed that in BHK cells ectopically expressing Ad receptors, Ad5 and Ad5/35 fiber knob-modified virus mainly target their cognate receptors CAR and CD46, respectively. 

### 2.3. CD46 Mediates Ad5/35 Gene Transduction in Bladder Cancer Cells

**Figure 4:**
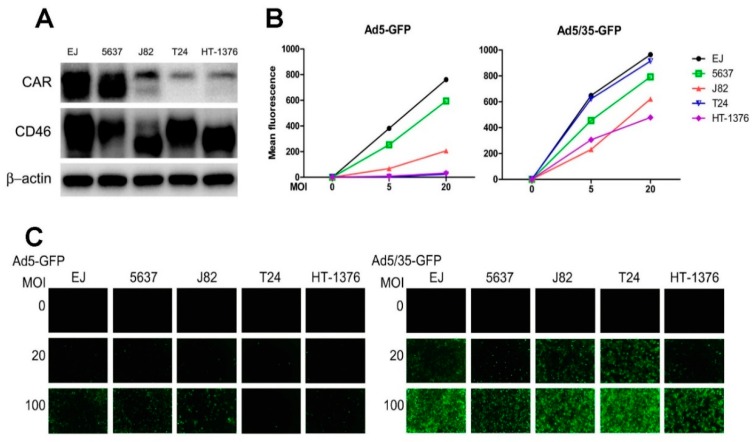
CD46 expression analysis and Ad5/35-mediated gene transduction in bladder cancer cell lines. (**A**) CAR and CD46 expression levels were analyzed in bladder cancer cell lines by Western blot analysis. (**B**) Transduction analyses of bladder cancer cell lines by flow cytometry following infection with different doses of Ad5-GFP or Ad5/35-GFP. (**C**) Transduction analyses of bladder cancer cell lines by fluorescence microscope following infection with the indicated multiplicities of infection (MOI) of Ad5-GFP or Ad5/35-GFP. At 24 h post-infection, GFP expression levels were monitored (20×).

To evaluate the adenoviral therapeutic efficiency of targeting either CAR or CD46 in bladder cancer cells, several human bladder cancer cells, including EJ, 5637, J82, T24, and HT-1376, were evaluated for their transduction susceptibility to Ad5 and Ad5/35. As shown in Figure 4A, CD46 was abundantly expressed in all five cancer cell lines, revealing multiple isoforms due to alternative splicing and polyadenylation [38,39]. CAR was prevalent in EJ and 5637 cells, but only weakly expressed in the other three cell lines. Accordingly, flow cytometry analysis revealed that Ad5-mediated GFP expression was mainly found in high CAR-expressing EJ and 5637 cells, with intermediate or very low levels in the other cells. In contrast, GFP transduction by Ad5/35 was high in all five cell lines (Figure 4B). A visual analysis of GFP expression following either Ad5 or Ad5/35 transduction in the five bladder cancer cell lines was performed using fluorescence microscopy, as shown in Figure 4C. The data suggest that Ad5/35-mediated GFP transduction is much more efficient in most bladder cancer cells. 

**Figure 5:**
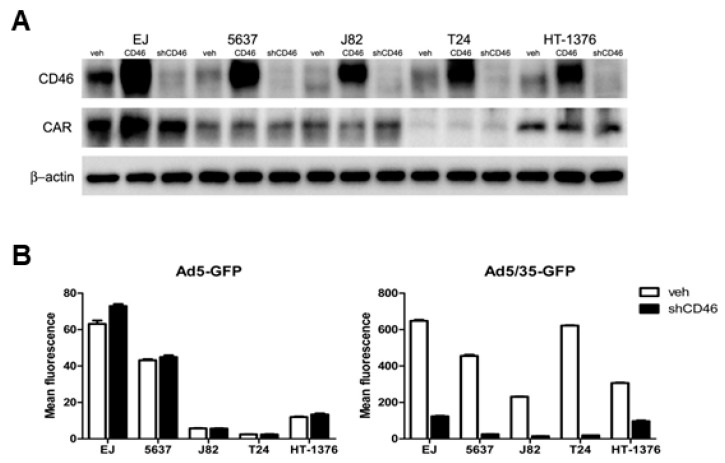
Gene transduction efficiency of Ad5 or Ad5/35 in CD46-altered bladder cancer cells. (**A**) Overexpression (CD46) and suppression (shCD46) of CD46 in bladder cancer cells relative to mock treated cells (veh) were confirmed by Western blot analysis. (**B**) Flow cytometry analysis of virus-mediated GFP transduction in CD46-suppressed and vehicle control cells was performed 24 h post infection. The error bars represent SEM. Statistics: B, *p* < 0.05 by two-way ANOVA.

In order to evaluate whether a change in CD46 expression levels affects Ad5/35-mediated gene transduction, lentiviruses were used to either overexpress CD46 or suppress endogenous CD46 in all five bladder cancer cells. The expression levels of CD46 and CAR in all of the generated stable cell lines were examined by Western blot analysis (Figure 5A). In general, neither overexpression nor suppression of CD46 affected CAR expression in the five cell lines, although there was a minor increase of CAR expression in CD46 overexpressing EJ cells. GFP transduction by either Ad5 or Ad5/35 was then accessed in the CD46-suppressed cells and viral vehicle-transduced control cells. Ad5/35-mediated GFP expression levels were dramatically reduced in all five CD46-suppressed cell lines (*p* < 0.05 by two-way ANOVA, right panel of Figure 5B), whereas Ad5-mediated GFP expression was not affected (left panel of Figure 5B). As CD46-overexpressing cell lines contain GFP-tagged CD46, we could not access similar transduction analysis.

**Figure 6:**
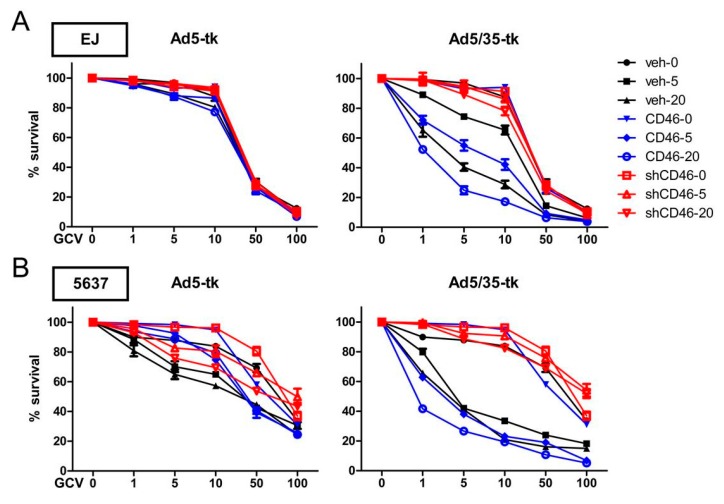

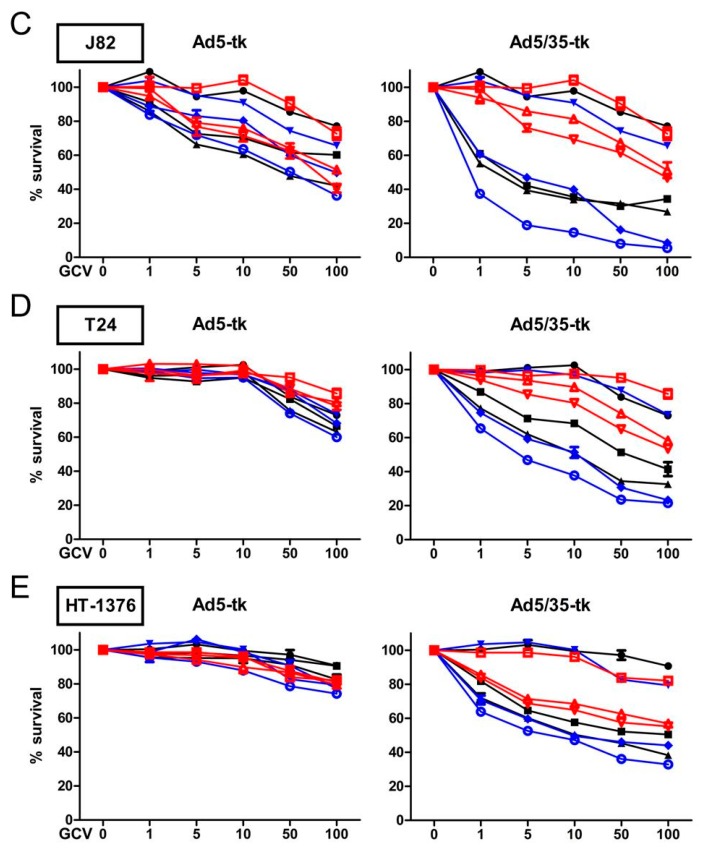
Ad5/35-tk-mediated enhancement of in vitro cytotoxicity in bladder cancer cells. CD46-overexpressing (CD46), CD46-suppressed (shCD46), or control EJ bladder cancer cells (veh) were seeded into 24-well plates and cultured overnight. Subsequently, the cells were either left untransduced or were transduced with Ad5-tk or Ad5/35-tk at the indicated MOIs of 5 and 20. The following day, increasing concentrations of GCV were added and an MTT in vitro proliferation assay was performed five days post-infection. Bladder cancer cells used in this experiment are EJ (**A**), 5637 (**B**), J82 (**C**), T24 (**D**), and HT-1376 (**E**). The error bars represent SEM. Statistics: *p* < 0.05 by two-way ANOVA.

In a next experiment, cytotoxicity assays using Ad5/35-tk in combination with GCV were performed in CD46-overexpressing, CD46-suppressed, or control cells derived from all five cancer cell lines. The altered expression of CD46 significantly modulated the Ad5/35-tk/GCV-mediated cytotoxic effect in a dose dependent manner (*p* < 0.001) (Figure 6A–E). Whereas Ad5/35-tk/GCV promoted cytotoxicity in CD46-overexpressing cells, CD46-supressed cells revealed strongly reduced sensitivity in cytotoxicity. Of note, control cells with intermediate CD46 levels also revealed intermediate cytotoxic sensitivity by Ad5/35-tk/GCV. In contrast, cytotoxic effect mediated by Ad5-tk/GCV was not affected by the CD46 expression status. These data suggest that our Ad5/35-targeted cancer therapeutic vector is highly effective for bladder cancer cells in vitro, in particular, when CD46 is expressed at high levels.

### 2.4. Efficacy of Ad5/35-Mediated Suicidal Gene Therapeutics Targeting CD46 in Human Bladder Cancer Cells In Vivo

**Figure 7:**
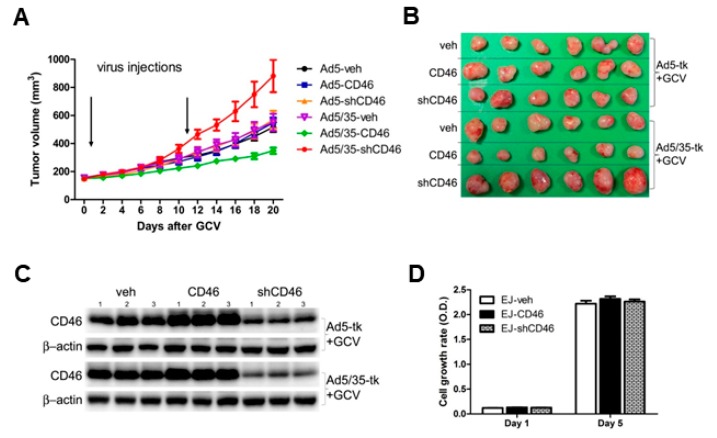
CD46 promotes Ad5/35-tk-mediated cytotoxicity of xenograft bladder tumor growth in vivo. (**A**) CD46 overexpressing (CD46), CD46 suppressed (shCD46), or control EJ bladder cancer cells (veh) were injected subcutaneously into nude mice. Intra-tumoral injections of Ad5/35-tk viruses (1.5 × 10^8^ plaque-forming units (PFU)) were performed twice at the indicated time points followed by intraperitoneal GCV injections (75 mg/kg) on days 2–12. Tumor growth was measured by a caliper at the indicated time points. (**B**) Representative photograph of tumor xenografts from mice treated with either Ad5-tk/GCV or Ad5/35-tk/GCV as described in (**A**). (**C**) Tumor tissue samples from the different tumor types and treatments were evaluated for CD46 expression by Western blot analysis. (**D**) Cell proliferation of each of different EJ cells was measured using the MTT assay. The error bars represent SEM. Statistics: A, *p* < 0.05 by two-way ANOVA.

To further investigate the in vivo efficacy of the CD46-targeted Ad5/35-tk/GCV, we used a xenograft tumor model in nude mice. For this, we chose the EJ bladder cancer cells as xenograft tumor, as they highly expressed both CAR and CD46 (Figure 5A). To induce tumors, we used 5 × 10^6^ CD46-overexpressing (CD46), CD46-suppressed (shCD46), or vehicle-transduced (veh) EJ cells were injected subcutaneously into the back of mice. When the tumor volumes reached approximately 150 mm^3^, 1.5 × 10^8^ PFU (plaque-forming units) of Ad5-tk or Ad5/35-tk was injected intratumorally on days 1 and 10. Subsequently, 75 mg/kg of GCV was administered intraperitoneally on day 2. Tumor-bearing mice were monitored and tumor size was measured every two days up to 20 days. As shown in Figure 7A, Ad5-tk/GCV did not significantly affect the in vivo growth of the three tumor types. In contrast, Ad5/35-tk/GCV affected tumor growth depending on the CD46 expression status. Compared with the vehicle-transfected group, Ad5/35-tk/GCV inhibited the tumor growth of CD46-overexpressing cells, but revealed reduced tumor cytotoxicity in CD46-suppressed cells (*p* = 0.005 by a repeated-measures ANOVA). Following Ad5/35-tk/GCV treatment, the average tumor volume was 48.55 ± 27.43 mm^3^ in the CD46-overexpressing group, 881.57 ± 141.72 mm^3^ in the CD46-reduced group, and 550.93 ± 74.39 mm^3^ in the vehicle group. A photographic image of isolated xenograft tumors from mice also showed that therapeutic effectiveness of Ad5/35-tk/GCV is tightly dependent on CD46 expression level (Figure 7B). A Western blot analysis of biopsy material derived from the three different tumor types revealed that CD46 expression in the xenograft tumors was not altered (Figure 7C), and also was not influenced by the treatment modality. To exclude that CD46 overexpression or suppression itself resulted in cell growth rate changes, we performed an in vitro cell proliferation assay (Figure 7D), which revealed that all three EJ tumor types had similar proliferation rates. In summary, our data suggest that modifying the adenoviral fiber knob to target CD46 gave rise to a highly effective way to target bladder cancer cells in vitro and in vivo, representing a promising therapeutic approach to treat bladder cancers.

## 3. Discussion

Tropism of Ads types is strongly determined by specific receptor interaction in cell culture infections, and most likely also influences in vivo infection behavior. Several virus receptors have been identified, and more recently, this information is increasingly translated into extending the virus type repertoire in order to improve gene therapy vector tropism. Treatment of diseases includes, in addition to cardiovascular and infectious disease, mainly cancer, which includes a plethora of cell variants. Ideally, for each of these applications, optimally targeted virus variants should be available. 

The best-studied adenoviral receptor is CAR, which is the major target host cell surface molecule for many adenovirus types, including Ad5, commonly utilized for gene therapy approaches [12,40,41,42]. CAR protein is expressed in numerous organ tissues including prostate, testis, pancreas, heart, brain, and intestine, but is poorly expressed in the lung, skeletal muscle, kidneys, placenta, ovaries, spleen, and leucocytes [14]. Although Ad5-mediated gene therapy works effectively with cells overexpressing CAR, expression of CAR is often down-regulated in many cancers, resulting in inefficient Ad-mediated therapeutic efficacy [43,44,45]. In this study, we found that CAR is generally overexpressed in human bladder tumor tissues and that CAR expression in human bladder cancer cell lines is clearly correlated with Ad5-mediated gene transduction. The expression of CAR in human bladder cancer cell lines was, however, highly variable; it was high in EJ and 5637 cells, but low in J82, T24, and HT-1376 cells. Accordingly, effective Ad5-mediated gene transduction was only seen in the high CAR-expressing EJ and 5637 cells (Figure 4). The CAR expression pattern, as well as CD46 expression pattern in bladder cancer cell lines, reported here are consistent with previous reports [46,47].

CD46 has been shown to be overexpressed in many primary cancers, as well as in tumor cell lines [19,48,49,50]. The role of this overexpression remains mostly unknown, but has been proposed to represent an immune evasion mechanism by which cancer cells protect themselves from complement dependent cytotoxicity [28,51]. We have previously shown that CD46 is highly expressed in cancers that originate from the colon and the prostate, being overexpressed in almost 60% of colon cancers [19]. In addition, CD46 expression was inversely correlated with the malignancy of colorectal cancers in colon cancers [19]. In this study, we showed that CD46 expression levels varied considerably among 59 tumor biopsies. In contrast, all five bladder cancer cell lines used here revealed high CD46 expression levels, including those with low CAR expression levels (J82, T24, and HT-1376 cells) (Figure 4A). Similarly, as found for colon cancers, the overexpression of CD46 in bladder cancers implied a negative correlation with tumor stage, tumor grade, and the risk group with better survival (Table 1, Figure 1 and Figure 2). In other words, CD46 is highly expressed in low risk cancer patients and is poorly expressed in high risk cancer patients. Currently, the molecular mechanisms leading to differential expression of CD46 during cancer cell progression is not understood. In head and neck squamous cancer cells, silencing MCPs did not render the cells susceptible to complement attack, suggesting that tumor cells can compensate for the lack of a single MCP by upregulating the expression of other complement restriction proteins [52].

As CD46 can also act as a receptor for various microbes, including species B adenoviruses such Ad35 [10,15,16], Ad35 vectors, or Ad5/35, chimeric vectors containing the Ad35 fiber gene have been generated and revealed strongly enhanced viral infectivity, with improved gene transduction and antitumor efficacy [10,53,54]. The latter adenovirus modification redirects the Ad5 vector to CD46, enhancing the ability of recombinant vectors to target CD46 overexpressing cells [19]. Here, we demonstrated that in all five bladder cancer cell lines tested, Ad5/35 had a much higher transduction efficacy and cytotoxicity compared with Ad5; this likely reflects the more abundant expression of CD46 in these bladder cancer cells (Figure 4). Ad5/35-medated activity was also dramatically increased in the low CAR-expressing cells, J82, T24, and HT-1376. Suppression of CD46 in all bladder cancer cells significantly decreased Ad5/35-mediated gene delivery, whereas it did not affect Ad5-mediated gene transduction (Figure 5). In agreement with these findings, the effects of Ad5/35 suicide gene therapy experiments correlated with the CD46 expression levels, which were manipulated by either overexpression or gene silencing (Figure 6). Likewise, in a tumor xenograft model in nude mice that was induced with cells manipulated to express different CD46 levels, there was a tight inverse correlation between CD46 expression and tumor growth following Ad5/35-tk treatment in combination with GCV (Figure 7). These results indicate that a recombinant chimeric Ad5/35 virus targeting CD46 can transduce bladder cancer cells more efficiently than a Ad5 virus targeting CAR. These results were also consistent with a previous study reporting limitations of Ad5-based gene therapy in vivo in ovarian cancer cells [55]. Partly because of the low viral transfection efficiency, conventional Ad5-based adenoviral cancer gene therapies might not lead to complete tumor eradication, and tumor cell growth resumes rapidly once treatment is stopped. In a baboon study, intravenous injection of Ad5/35 also had a better safety profile than conventional Ad5 [56]. In line with our results, these data suggest that multiple and continuous intratumoral injection with viruses revealing enhanced transduction efficacy might be required to improve adenovirus-mediated gene therapy.

## 4. Materials and Methods 

### 4.1. Immunohistochemistry

Clinical human bladder cancer samples were obtained from patients undergoing surgical resection at Chonnam National University Hwasun Hospital (Hwasun, Korea). Immunohistochemistry was performed and evaluated as previously described [19].

### 4.2. Cell Lines and Cell Culture

Human bladder cancer cell lines were purchased from American Type Culture Collection (ATCC, Manassas, VA, USA), including 5637 (HTB-9), J82 (HTB-1), T24 (HTB-4), and HT-1376 (CRL-1472). The EJ human bladder cancer cell line was obtained as a gift from Chungbuk National University Department of Urology. The 293T (CRL-3216) cell line was also from ATCC. Baby hamster kidney (BHK) cells expressing CAR and CD46 cells have been described previously [10,42]. The 293T, EJ, and BHK cells lines were routinely cultured in Dulbecco’s modified Eagle’s medium (DMEM; Welgene, Korea) supplemented with 5% heat-inactivated fetal bovine serum (FBS; Welgene, Korea) and 1% penicillin/streptomycin (Gibco, Life Technologies, Grand Island, NY, USA). The other cell lines were maintained in complete Roswell Park Memorial Institute-1640 medium (RPMI, Welgene, Korea) with addition of 5% FBS and 1% penicillin/streptomycin. All cells were cultured at 37 °C/5% CO_2_. Lentiviral vector pBlasti-eGFP-CD46 was produced in 293T cells as described [57] and used for CD46 overexpression studies in bladder cancer cells. Lentiviral particles with CD46 shRNA were purchased from Santa Cruz Biotechnology (Santa Cruz, CA, USA) and transfected into cells per the manufacturer’s protocol. For constructing control cells, either pBlsti or pBlasti-GFP vectors were used. Cells were cultured in media with blasticidin (10 µg/mL) for selection over three weeks and positive clones were confirmed by Western blotting.

### 4.3. Viruses

Ad5 CMV-GFP and Ad5/35CMV-GFP were constructed as previously described [58]. Viral production and titer were done as previously described [59]. Briefly, the viral vector was transfected into 293 cells with lipofectamine (Gibco, Rockville, MD, USA) and selected clones were propagated in 293 cells, purified by CsCl_2_ gradient centrifugation, and dialyzed against 10 mM Tris-HCl (pH 7.5)/1 mM MgCl_2_ buffer supplemented with 10% glycerol. The viral titer was determined by measuring the optical density at 260 nm after lysing viral particles in 5% sodium dodecyl sulfate (SDS). 

### 4.4. Immunoblotting Analysis

Total protein lysate (20 μg) was separated on a 10% SDS-polyacrylamide gel and then transferred to Immobilon–P membrane (Millipore, Billerica, MA, USA). The membranes were blocked with 5% non-fat skim milk before being incubated with primary antibodies at 4 °C overnight. Antibodies against β-actin were purchased from Sigma-Aldrich Inc. (St. Louis, MO, USA). Anti-CAR and anti-CD46 antibodies were from Santa Cruz Biotechnology (Santa Cruz, CA, USA). After being incubated for 1 h with secondary antibodies, proteins were detected and analyzed by Immobilon (Millipore, Billerica, MA, USA) and ChemiDOC ^TM^ MP Imaging System (Bio-Rad, Hercules, CA, USA).

### 4.5. FACScan Analysis and Immunofluorescence

Cells were seeded into six-well culture dishes and cultured overnight at 37 °C, 5% CO_2_. The next day, cells were infected with Ad5-GFP or Ad5/35-GFP virus at various multiplicities of infection (MOI 0, 5, 20, and 100) for 2 h. Then, viruses were removed by replacing with fresh media. At 24 h after infection, the cells were harvested by cell scrapers and washed twice with phosphate buffered saline (PBS). All cyto-fluorometric determinations were carried out using a FACSCalibur flow cytometer (BD Biosciences, San Jose, CA, USA). The data were analyzed using Kaluza Analysis software (Beckman Coulter, Inc., Brea, CA, USA). Immunofluorescence was performed and evaluated as previously described [19]. 

### 4.6. Cell Killing Assay

To evaluate the in vitro cell killing efficacy of thymidine kinase-expressing viruses (Ad5-tk and Ad5/35-tk) in combination with ganciclovir (GCV, JHP Pharmaceuticals LLC., Rochester, MI, USA), 1 × 10^4^ cells per well were seeded into 24-well plates. The following day, the medium was changed to 500 μL of DMEM/ bovine serum albumin (BSA) (20 mg/mL) containing Ad5-tk or Ad5/35-tk at various MOIs (0, 5, and 20). After 2 h of infection, viruses were removed by replacing with fresh media. At 24 h post infection, serial concentrations (0, 1, 5, 10, 50, and 100 μg/mL) of GCV were added to the wells for four days. Cell viability was assessed using the MTT assay. The absorbance was measured at 570 nm using a microplate reader with SOFTmax PRO software (Molecular Devices, Sunnyvale, CA, USA). 

### 4.7. Animal Studies

Six-week-old nude mice (Central Lab Animal Inc., Seoul, Korea) were maintained in a specific pathogen-free facility. All animal experiments conformed to Chonnam National University Animal Research Committee protocols. Mice were randomly allocated to six different groups (six mice per each group). Control EJ-wt, EJ-CD46, and EJ-shCD46 cells were injected subcutaneously into the flanks of mice (5 × 10^6^ cells for each mouse). When the tumor volumes reached approximately 150 mm^3^, 1.5 × 10^8^ PFU of Ad5-tk or Ad5/35-tk were infected by intratumoral injection on days 1 and 10, followed by 75 mg/kg of GCV administered intraperitoneally on days 2–15. Every two days, tumor size was measured and calculated using the following equation: (length × width^2^)/2.

### 4.8. Statistical Analysis

Statistical analysis was performed as previously described [19]. In brief, SPSS 21.0 software (IBM, Chicago, IL, USA) was used to analysis and all data were presented as mean ± standard error (SEM). Data were analyzed using a one-tailed Student’s *t*-test. Statistical significance was achieved when the *p* value was less than 0.05.

## 5. Conclusions

In summary, our data indicate that use of Ad5/35 fiber chimeric gene therapy vectors re-directed to CD46 provides an effective approach to enhance viral gene transduction efficacy in CAR-deficient cancers, including a subset of bladder cancers. However, because of the inverse correlation between CD46 expression and cancer malignancy in bladder cancer patients, careful consideration needs to be given to select patients for effective adenoviral cancer gene therapy.

## Figures and Tables

**Table 1:** Correlation between CD46 expression and clinico-pathological features in patients with bladder cancer.

Variables	All Patients (*n* = 59)	Low CD46 (*n* = 31)	High CD46 (*n* = 28)	*p*-Value *
**Age (IQR)**	72.7 (63.4–78.3)			
≤73	30 (50.8)	16 (51.6)	14 (50.0)	0.902
>73	29 (49.2)	15 (48.4)	14 (50.0)	
**Sex (*n*, %)**				
Male	46 (78.0)	27 (87.1)	19 (67.9)	0.075
Female	13 (22.0)	4 (12.9)	9 (32.1)	
**BMI (kg/m^2^, IQR)**	24.0 (22.0–26.1)			
<25	37 (62.7)	18 (58.1)	19 (67.9)	0.437
≥25	22 (37.3)	13 (41.9)	9 (32.1)	
**ASA score**				
1	6 (10.2)	2 (6.5)	4 (14.3)	0.503
2	47 (79.7)	25 (80.6)	22 (78.6)	
3	6 (10.2)	4 (12.9)	2 (7.1)	
**DM (*n*, %)**	12 (20.3)	6 (19.4)	6 (21.4)	0.843
**Hypertension (*n*, %)**	32 (54.2)	21 (67.7)	11 (39.3)	0.028 **
**Tumor size (cm, IQR)**	1.5 (1.0–3.0)			
**T stage (*n*, %)**				
Ta	34 (57.6)	14 (45.2)	20 (71.4)	0.017 **
T1	23 (39.0)	17 (54.8)	6 (21.4)	
CIS	2 (3.4)	0 (0)	2 (7.1)	
**Grade (*n*, %)**				
Low	32 (54.2)	12 (38.7)	20 (71.4)	0.012 **
High	27 (45.8)	19 (61.3)	8 (28.6)	
**Multifocality (*n*, %)**	21 (35.6)	9 (29.0)	12 (42.9)	0.268
**Concomitant CIS (*n*, %)**	3 (5.1)	1 (3.2)	2 (7.1)	0.494
**EORTC risk group (*n*, %)**				
Low	21 (35.6)	8 (25.8)	13 (46.4)	0.042 **
Intermediate	11 (18.6)	4 (12.9)	7 (25.0)	
High	27 (45.8)	19 (61.3)	8 (28.6)	
**Intravesical Chemotherapy (n, %)**				
No	42 (71.2)	22 (71.0)	20 (71.4)	0.129
BCG	14 (23.7)	9 (29.0)	5 (17.9)	
Adriamycin	3 (5.1)	0 (0)	3 (10.7)	
**Recurrence (*n*, %)**	26 (44.1)	16 (51.6)	10 (35.7)	0.219

*, Pearson’s χ^2^ test was used; **, statistically significant; IQR, interquartile range; BMI, body mass index; ASA score, American society of anesthesiologists score; DM, diabetes mellitus; CIS, carcinoma in situ; EORTC, European organisation for research and treatment of cancer; BCG, bacille Calmette-Guerin.
